# Accuracy of inverse treatment planning on substitute CT images derived from MR data for brain lesions

**DOI:** 10.1186/s13014-014-0308-1

**Published:** 2015-01-10

**Authors:** Joakim H Jonsson, Mohammad M Akhtari, Magnus G Karlsson, Adam Johansson, Thomas Asklund, Tufve Nyholm

**Affiliations:** Department of Radiation Sciences, Umeå University, Umeå, SE-901 87 Sweden

**Keywords:** Radiotherapy, Treatment planning, MRI, Substitute CT, s-CT

## Abstract

**Background:**

In this pilot study we evaluated the performance of a substitute CT (s-CT) image derived from MR data of the brain, as a basis for optimization of intensity modulated rotational therapy, final dose calculation and derivation of reference images for patient positioning.

**Methods:**

S-CT images were created using a Gaussian mixture regression model on five patients previously treated with radiotherapy. Optimizations were compared using *D*_max_, *D*_min_, *D*_median_ and *D*_mean_ measures for the target volume and relevant risk structures. Final dose calculations were compared using gamma index with 1%/1 mm and 3%/3 mm acceptance criteria. 3D geometric evaluation was conducted using the DICE similarity coefficient for bony structures. 2D geometric comparison of digitally reconstructed radiographs (DRRs) was performed by manual delineation of relevant structures on the s-CT DRR that were transferred to the CT DRR and compared by visual inspection.

**Results:**

Differences for the target volumes in optimization comparisons were small in general, e.g. a mean difference in both *D*_min_ and *D*_max_ within ±0.3%. For the final dose calculation gamma evaluations, 100% of the voxels passed the 1%/1 mm criterion within the PTV. Within the entire external volume between 99.4% and 100% of the voxels passed the 3%/3 mm criterion. In the 3D geometric comparison, the DICE index varied between approximately 0.8-0.9, depending on the position in the skull. In the 2D DRR comparisons, no appreciable visual differences were found.

**Conclusions:**

Even though the present work involves a limited number of patients, the results provide a strong indication that optimization and dose calculation based on s-CT data is accurate regarding both geometry and dosimetry.

## Background

For several common patient groups in the radiotherapy clinic, there is strong support within the scientific community for using magnetic resonance (MR) imaging as the primary imaging modality when defining the target volume. Examples of such patient groups are those with prostate [1], brain [2], head and neck [3] and cervical cancers [4]. Treatment planning, including inverse planning optimization, dose calculations and generation of reference images for patient positioning are still dependent on the access to computed tomography (CT) images of the patient due to the electron density information content and excellent contrast between bone and soft tissue. However, a workflow in which the target volume is defined on MR images and the treatment is planned on CT data is dependent on the ability to align these images in the same coordinate system, i.e. image registration. The literature indicates that multi-modal image registration on actual patient data is associated with uncertainties of clinically relevant magnitude, affecting the geometric accuracy of the treatment systematically [5]. Therefore, it has been suggested to use MR data exclusively for the planning of the treatment [6-9] to avoid such spatial uncertainties. In order to use MR data exclusively, some form of conversion of MR image intensities into values that resemble Hounsfield units (HU) is necessary, since dose calculations rely on the connection between HUs and electron densities. Several different methods have been proposed, and the reported dosimetric results of calculations based on MR are generally good [10-13]. The early work within the area was based on either manual delineation of relevant anatomical structures and assigning them bulk densities, which is highly time consuming [10,13,14], or using a single bulk density for the entire anatomy, i.e. that of water or mixture of muscle and adipose tissue. Such single density conversions compromises the dosimetric accuracy to a higher degree than multiple bulk density assignment and makes it impossible to generate adequate reference images for positioning [13]. The introduction of MR imaging with ultra-short echo-times (UTE), enabled automatic separation of bone and air on a voxel-by-voxel level [15,16]. UTE images with different contrasts, combined with a statistical method associating the intensities in the MR images with HUs has been shown to provide a CT-like image without manual procedures [17]. These CT-like images can be used as a substitute CT (s-CT) for MR only treatment planning [18], or as basis for attenuation correction of positron emission tomography (PET) data acquired with a combined PET/MR scanner [19]. In the present pilot study we evaluate the performance of an s-CT image, derived from MR data using a specific method, as a basis for optimization of intensity modulated rotational therapy, final dose calculation and derivation of reference images for patient positioning.

## Methods

### Study design

**Figure 1:**
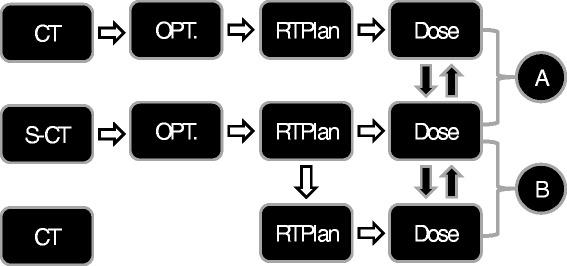
**Overview of study design.** Dose distributions from separate optimizations on s-CT and CT were compared in **(A)**. Doses based on the s-CT optimization but recalculated on the CT were compared in **(B)**.

The dosimetric comparisons in the present study separated the optimization and dose calculation steps as illustrated in Figure 1. To compare the optimizations, plans with the exact same target volume, organs at risk and optimization constraints were derived based on CT and s-CT data and the dose distributions were compared (A). To compare the dose calculations, the plan optimized on the s-CT was copied to the CT study, and the final dose calculations based on the same plan were compared (B).

### Patient data and imaging

**Figure 2:**
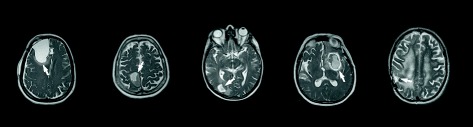
**An overview of the five patients included in the study.** The figure shows selected slices from a T2 weighted sequence. Lesions are marked with white arrows.

The study was approved by the institutional ethical committee. Five consecutive patients with intracranial tumors (glioblastoma multiforme) referred to radiotherapy were included in the study. This patient group underwent both MR and CT examinations as part of their standard preparation for radiotherapy, see Figure 2 for an overview. For the five patients included in the study, the MR examination was extended with two dual-echo UTE image acquisitions, with echo-times of 0.07 and 3.42 ms. In the first acquisition, a nominal flip-angle of 10 degrees was used and the second had a nominal flip-angle of 60 degrees. The UTE imaging was performed with isotropic 1.3 × 1.3 × 1.3 mm^3^ voxels. The MR examinations were performed using a 1.5 T Siemens Espree scanner and a standard 8 channel head coil. All MR data was distortion corrected using the 3D distortion correction provided by the vendor [20,21]. CT images were acquired using a Siemens Emotion 6 (three patients) and a GE Discovery 690 (two patients), both calibrated for use within radiotherapy. Tube voltage was 130 kV for the Emotion 6 and 120 kV for Discovery 690. Slice thicknesses varied between 2.5 mm and 3.75 mm and in-plane resolution between 0.5 mm and 1.4 mm.

### Derivation of s-CT

The s-CT was estimated using the method described by Johansson et al. [16], with the image data from the two dual-echo UTE scans and a T2-weighted 3D sequence (Siemens SPACE). The patients in the present study were not imaged in treatment position in the MR scanner. Therefore, an off-line image registration was performed to align the CT and s-CT in the same coordinate system using a rigid Mattes mutual information image algorithm from the Insight Toolkit (ITK). The result of each registration was verified by visual inspection.

### Treatment planning

Oncentra version 4.3 (Elekta, Stockholm, Sweden), was used to plan the treatments. A radiation oncologist (T.A) defined the target volume (GTV) and the organs at risk (chiasma, brainstem, pituitary, right and left lens, right and left opticus nerve) for each patient. The same structure set was associated with both the CT and s-CT via the previously described image registration, with the exception of the outer contour which was derived individually for both image sets. The optimization of the treatments was performed based on both the s-CT and the CT using the exact same optimization constraints and objectives, see Table 1. For the comparison of the optimization results, the dose volume histograms (DVHs) were exported for the s-CT and CT plan. To compare the performance of the final dose calculation on the two image sets, the plan optimized based on the s-CT was transferred to the CT data set, recalculated and the dose distribution was exported. Dose calculations were performed using a pencil beam algorithm, and a dose matrix with 1.0 × 1.0 × 1.0 mm^3^ resolution.

**Table 1:** **The optimization objectives used in the study**

**Structure**	**Objective**	**Weighting factor**
GTV	Min dose > 58	3000
PTV	Min dose > 57	3000
	Max dose < 63	
Left lens	Max dose < 5	1
Right lens	Max dose < 5	1
Left opticus	Max dose < 5	1
Right opticus	Max dose < 5	1
Chiasma	Max dose < 50	1
Brain stem	Max dose < 60	1
Pituritary gland	Max dose < 40	1
External	Surrounding dose falloff: 60 to 30 Gy in 10 mm	1000

### Dosimetric evaluation

For the evaluation of the optimization results, the *D*_max_, *D*_min_, *D*_median_ and *D*_mean_ doses to the PTV and OAR’s were compared between plans optimized on CT and s-CT image data. The evaluation of the dosimetric accuracy of the s-CT based calculations was performed by copying the plan optimized on the s-CT to the CT study, calculating the resulting dose and comparing dose matrices via gamma analysis [18] using acceptance criteria of 3%/3 mm and 1%/1 mm. Although measurement errors are absent, small geometrical deviations may be present due to registration inaccuracies or geometric distortions in the MR images.

### Geometric evaluation

The geometric evaluation of the s-CT was performed for the 2D case, i.e. s-CT based digitally reconstructed radiographs (DRRs), as well as in 3D. Patient positioning using 2D images and DRRs for intra-cranial treatments is often performed by manual alignment of bony structures visible on 2D x-ray projections. The verification of the geometric accuracy of s-CT based DRRs therefore focused on the geometric accuracy of the depiction of these structures. DRRs for the CT and s-CT were generated, and the structures commonly used for patient positioning were delineated on the s-CT based DRR. These delineations were then overlaid on the CT based DRR and the result was evaluated by visual inspection.

When using cone beam CT (CBCT) for positioning of the patient, automatic 3D registration is often the method of choice. Given the poor soft tissue contrast of CT and CBCT, this registration will be dominated by the high contrast bony structures. Therefore, to evaluate the geometric accuracy of the s-CT for 3D positioning, the bony structures were identified on both CT and s-CT data using an image intensity threshold of 400 HU and the dice similarity index was calculated, for individual slices as well as for the entire volume.

## Results

### Dosimetric comparison - optimization

The differences between the plans optimized based on CT and s-CT was in general very small both for target and organs at risk, as can be seen in Table 2. None of the patients fully met all optimization criteria. For the patients (n = 3) which did not reach the 58 Gy minimum criterion for the GTV in at least one of the two optimizations, the average minimum dose was 57.2 for the CT optimization and 57.7 for the s-CT optimization. For the patients which did not fully reach the PTV related criteria (n = 5), the average minimum dose to the PTV was 54.2 and 54.3 Gy and the average maximum dose was 63.7 and 63.5 Gy, for the CT and s-CT optimizations respectively. Only one of the patients failed to fulfill any optimization criteria for the OAR. This patient exceeded the criteria for the brain stem, pituitary gland, chiasma and left lens. For all these OARs, the result was slightly closer to fulfilling the criteria in the s-CT optimization. The relative dose differences in the OARs were greatest in the optic nerves, most likely due to the fact that the optic nerve passes in proximity to the nasal cavity which is very difficult to classify using MRI in combination with the low doses to the optic nerve, i.e. a small absolute difference in dose will yield a large percentage error. However, none of these differences was statistically significant.

**Table 2:** **Dose comparison results**

	**Diff.** ***D***_**min**_ **(%)**	**Diff.** ***D***_**max**_ **(%)**	**Diff.** ***D***_**median**_ **(%)**	**Diff.** ***D***_**mean**_ **(%)**
PTV	0.3 (1.6)	−0.3 (0.6)	−0.1 (0.1)	0.0 (0.0)
GTV	0.8 (1.0)	0.5 (0.6)	0.3 (0.8)	0.3 (0.7)
Brainstem	−5.9 (7.9)	−0.4 (4.0)	1.6 (3.7)	−0.4 (1.9)
Pituitary gland	−0.9 (4.0)	0.9 (3.5)	0.7 (3.5)	0.1 (3.4)
Right lens	4.8 (8.6)	0.4 (6.4)	2.3 (7.8)	2.3 (7.7)
Left lens	4.6 (2.0)	−5.0 (24.8)	5.2 (4.4)	5.1 (4.0)
Right opticus	1.2 (5.6)	0.6 (4.7)	1.6 ( 4.0)	2.3 (5.0)
Left opticus	4.2 (4.3)	5.1 (11.6)	−2.2 (11.6)	5.0 (6.3)
Chiasma	−2.1 (3.1)	−0.7 (3.5)	−0.7 (3.3)	−1.0 (3.6)

Legend: Average differences between D_max_, D_min_, D_median_ and D_mean_ dose for plans optimized on CT and s-CT. The standard deviation calculated based on the five patients are presented within brackets.

### Dosimetric comparison – dose calculation

**Figure 3:**
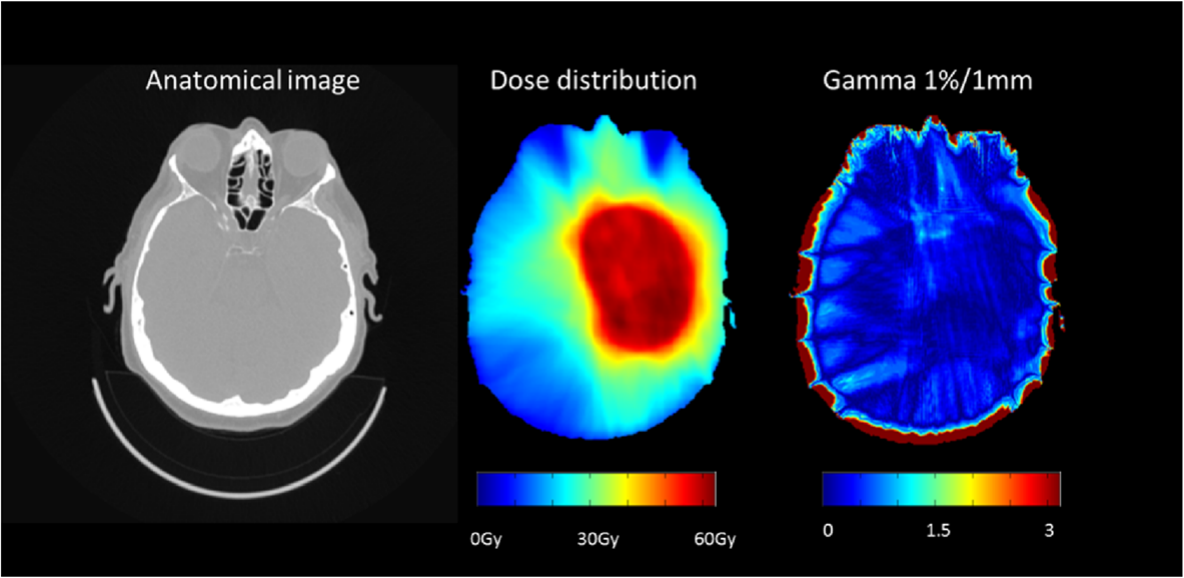
**Anatomical CT image, dose distribution and gamma map for a representative slice of the patient with the largest observed dosimetric errors.** The high dose region extends into the ethmoidal and sphenoidal sinuses.

The plan optimized on the s-CT data was copied to the CT study and final dose calculation was performed based on both s-CT and CT, and the results were compared. 100% of the voxels passed the 1%/1 mm criterion within the PTV. Within the entire external volume between 99.4% and 100% of the voxels passed the 3%/3 mm criterion. For the 1%/1 mm criterion between 93.7% and 98% of the voxels passed. An example of a gamma map is shown in Figure 3.

### Geometric comparison 2D

**Figure 4:**
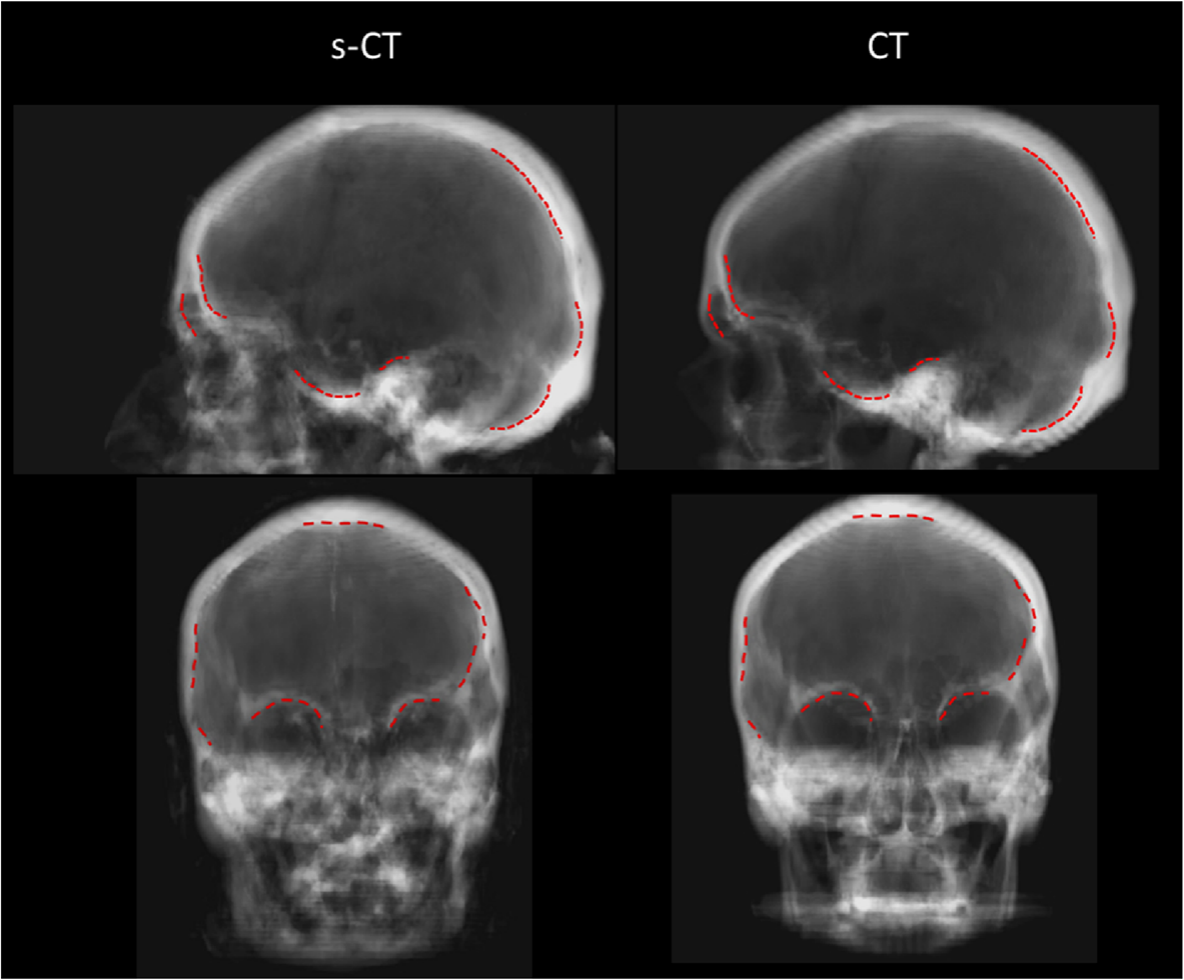
**Lateral and anterior projections of the s-CT data (left) and CT data (right) for a representative patient.** Structures deemed relevant for patient positioning are displayed using dashed red lines. The structures were delineated on the s-CT DRR and copied to the CT DRR. No obvious geometric differences between the two DRRs can be detected.

The s-CT based DRRs had a more blurred appearance compared to their CT based counterparts. This was especially pronounced in areas where the projections passed through internal air cavities. The nasal septum was not visible on the s-CT DRR in the same way as in the CT based DRR. For the bony structures relevant for patient positioning at treatment, the different DRRs were deemed clinically equivalent. There was no detectable geometrical difference except for the slight blurring. Examples of s-CT and CT based DRRs are shown in Figure 4.

### Geometric comparison 3D

**Figure 5:**
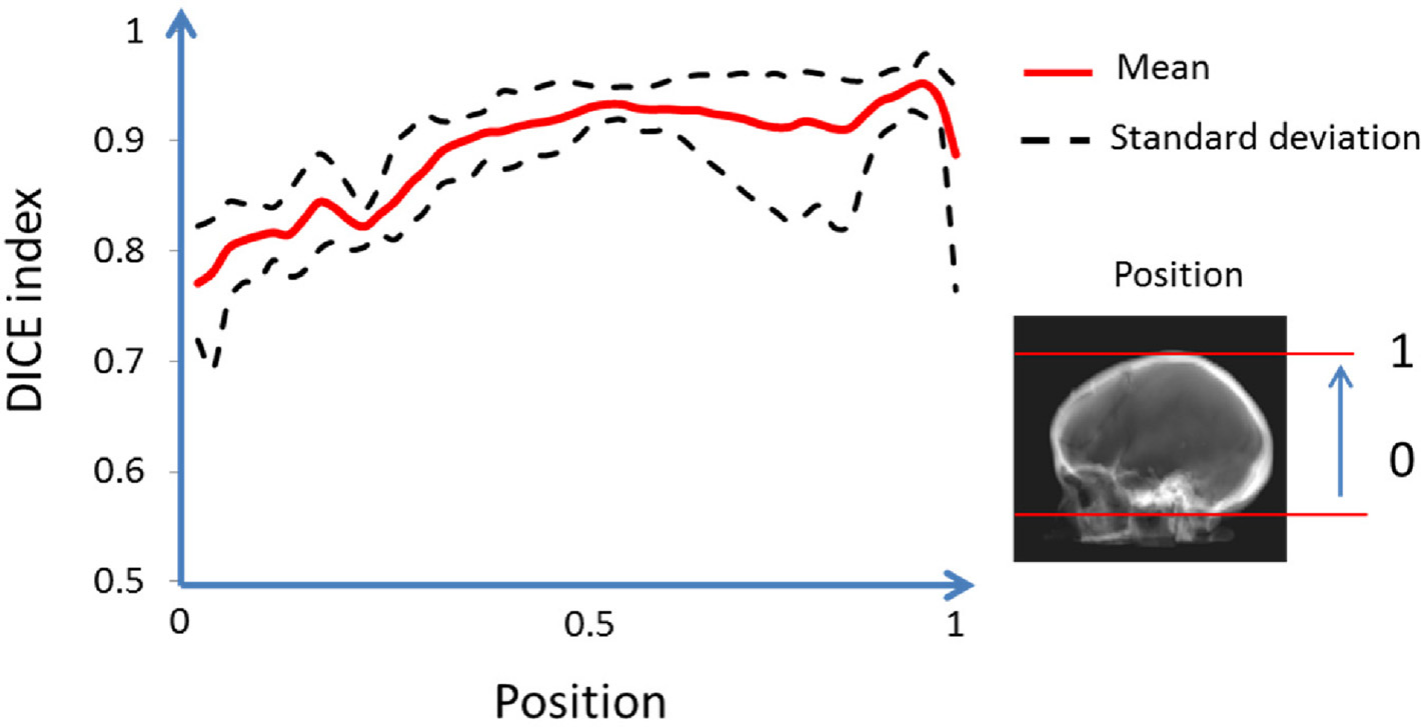
**The dice index as function of the position in the z-direction.** A 400 HU threshold was used for both CT and s-CT.

**Figure 6:**
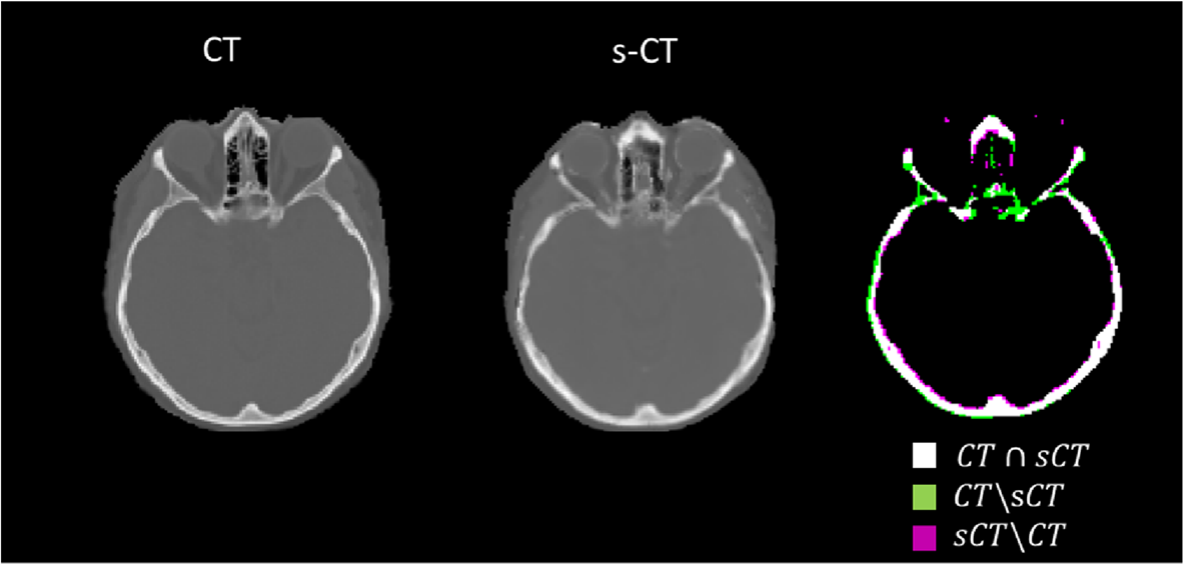
**CT, s-CT and illustration of overlapping bony anatomy.** The DICE index for the slice on display was 0.81.

The DICE index distribution for bone with density above 400 HU as a function of the position in the z-direction in the skull is displayed in Figure 5. The agreement between s-CT and CT increases cranially, with a DICE index around 0.8 at the level of the caudal cerebellum to around 0.9 at the level of central cerebrum and above. Figure 6 shows a representative slice of the CT, the s-CT and a difference map. It is clear that the posterior nasal cavities are the most problematic with misclassifications in both directions.

## Discussion

This pilot study evaluated the feasibility of using CT equivalent data (s-CT) derived from MR images for optimization, final dose calculation and generation of reference images for patient positioning at treatment. Even though the patient material was very limited (only five patients), the results did not suggest that neither the quality of the optimizations nor the final dose calculation are compromised using the s-CT data as input for the treatment planning. We therefore conclude that treatment planning based on s-CT and CT may be equivalent for intracranial lesions, provided that the MR acquisition was successful. MR imaging is known to be sensitive to motion artifacts and there is some apprehension that the quality of the s-CT may be insufficient for a currently unknown fraction of the patients. A larger study is needed to investigate this and to serve as basis for the development of a patient specific quality control method for the s-CT.

The DRRs based on s-CT data are very similar to the golden standard – CT based DRRs. The important structures are geometrically correct and are easy to find in the s-CT based DRRs. In 3D, a DICE coefficient between 0.8 and 0.9 was found, with the higher value for the cranial half of the skull. This compares well with other published methods to segment the skull from MR data. Dogdas et al. presented a method to segment the skull based on a single high resolution MR scan, resulting in a DICE coefficient of on average 0.75 [22]. Wagenknecht et al. evaluated an automatic segmentation method based on 3D MP-RAGE or other T1 weighted MR data and presented DICE coefficients between 0.7 and 0.8 in the cranial part of the skull with lower values caudally, using a Hounsfield threshold of 500 HU [23].

## Conclusion

In conclusion, the results of our study provide experimental proof that it is feasible to use MR based s-CTs for optimization of the treatment plans in Oncentra. The differences between plans optimized on s-CT and CT are very small, well below what can be considered clinically important, both in terms of target coverage and avoidance of risk organs. Recalculations of s-CT based treatment plans using CT data revealed very small dose calculation errors. Even though the present work involves a limited number of patients, it provides a strong indication that optimization and dose calculation based on s-CT data is safe and may be used in clinical practice. Other experimental studies on this method with larger number of patients would be of major interest to improve our knowledge and serve as a basis for development of a quality assurance strategy.
